# Genetic, Epigenetic, and HPLC Fingerprint Differentiation between Natural and Ex Situ Populations of *Rhodiola sachalinensis* from Changbai Mountain, China

**DOI:** 10.1371/journal.pone.0112869

**Published:** 2014-11-11

**Authors:** Wei Zhao, Xiaozheng Shi, Jiangnan Li, Wei Guo, Chengbai Liu, Xia Chen

**Affiliations:** 1 National and Local United Engineering Laboratory for Chinese Herbal Medicine Breeding and Cultivation, School of Life Science, Jilin University, Changchun, Jilin, China; 2 Key Laboratory for Molecular Enzymology and Engineering, the Ministry of Education, Jilin University, Changchun, Jilin, China; 3 School of Life Science, Jilin University, Changchun, Jilin, China; 4 Institute of Botany, Changbai Mountain Academy of Sciences, Erdao, Jilin, China; The National Orchid Conservation Center of China; The Orchid Conservation & Research Center of Shenzhen, China

## Abstract

*Rhodiola sachalinensis* is an endangered species with important medicinal value. We used inter-simple sequence repeat (ISSR) and methylation-sensitive amplified polymorphism (MSAP) markers to analyze genetic and epigenetic differentiation in different populations of *R. sachalinensis*, including three natural populations and an *ex situ* population. Chromatographic fingerprint was used to reveal HPLC fingerprint differentiation. According to our results, the ex situ population of *R. sachalinensis* has higher level genetic diversity and greater HPLC fingerprint variation than natural populations, but shows lower epigenetic diversity. Most genetic variation (54.88%) was found to be distributed within populations, and epigenetic variation was primarily distributed among populations (63.87%). UPGMA cluster analysis of ISSR and MSAP data showed identical results, with individuals from each given population grouping together. The results of UPGMA cluster analysis of HPLC fingerprint patterns was significantly different from results obtained from ISSR and MSAP data. Correlation analysis revealed close relationships among altitude, genetic structure, epigenetic structure, and HPLC fingerprint patterns (*R^2^* = 0.98 for genetic and epigenetic distance; *R^2^* = 0.90 for DNA methylation level and altitude; *R^2^* = –0.95 for HPLC fingerprint and altitude). Taken together, our results indicate that ex situ population of *R. sachalinensis* show significantly different genetic and epigenetic population structures and HPLC fingerprint patterns. Along with other potential explanations, these findings suggest that the ex situ environmental factors caused by different altitude play an important role in keeping hereditary characteristic of *R. sachalinensis*.

## Introduction

Plant is one of the most important resources that human being depend on, because of the direct benefits to humans that arise from its exploitation in new agricultural and horticultural crops. The development of medical drugs and the pivotal role played by plants are the functions of all natural ecosystems [1]. Plant diversity conservation has become a topic of common concern in the world. Ex situ conservation is the process of protecting an endangered species of plant or animal outside its natural habitat. In situ conservation is the best method of plant diversity conservation. But unfortunately, in situ conservation is not an option in many cases. The natural habitat of many species have already been completely degradation or loss caused by human over disturbance, and those of many others have been so reduced in size and so fragmented that the species are in danger of extinction [2]. Even when habitat preservation can be practical, it frequently requires the reintroduction of species. In such cases, ex situ conservation is needed to avoid species extinction in nature [1]. Ex situ conservation plays the most important role in the plant conservation especially through Botanical garden.

In recent years, some studies of population genetic structure in ex situ conservation species indicated that they may exist in a potential genetic risk, such as the loss of genetic diversity, inbreeding depression, outbreeding depression and genetic adaptation [3], [4], [5], [6]. For ex situ conservation of endangered plants, due to environmental changes, species may generate inheritable variation in phenotype, resistance, life history etc. Those changes will lead to changes in species evolution route and a certain difficulty to future the reintroduction of species [7], [8]. Consequently, the amount of genetic variation holding in the captive population is critical to assure the success of ex situ conservation and subsequent releasing. In addition, evaluation of genetic status for ex situ conservation species is also very important.

*Rhodiola sachalinensis* A. Boriss. is a perennial herbaceous plant in the family Crassulaceae. In China, this species is mainly distributed in alpine zones of Changbai Mountain and the southeastern Zhangguangcai Mountain, where plants are found at altitudes ranging from 1,700 to 2,500 m [9]. This species is rare with a fragment distribution. Due to heavy disturbance by human activities, it is now endangered, and has been listed as an endangered plant species in the Chinese Plant Red Book [10]. *R. sachalinensis* is a medicinal plant, containing the effective component of salidroside in roots and rhizomes, and has a reputation for stimulating the nervous system, decreasing depression, relieving fatigue, resisting anoxia and microwave radiation, preventing high altitude sickness, and improving sleep [11], [12], [13].

**Figure 1:**
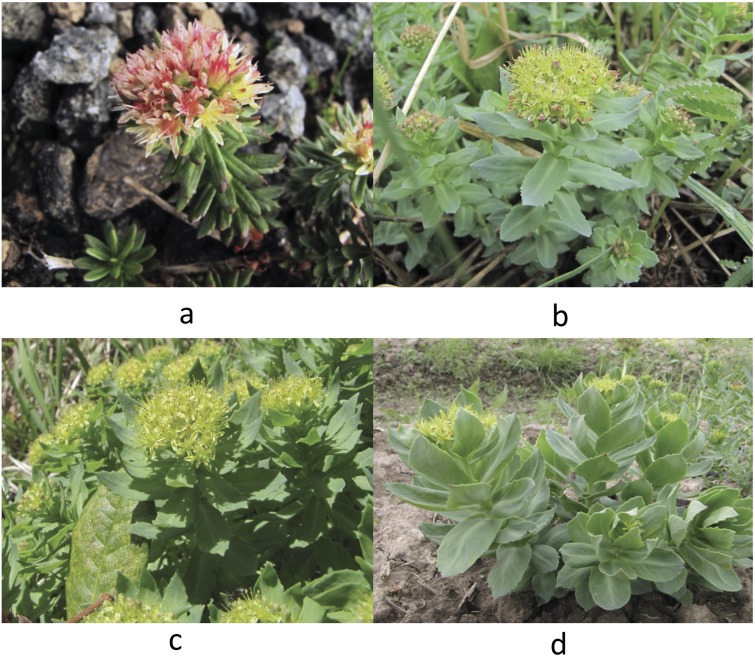
Morphological differences exhibited by *R. sachalinensis* at different altitudes. (a) Growth at 2,594 m; (b) Growth at 2,293 m; (c) Growth at 2,007 m; (d) Growth at 763 m (cultivated population).

From the year 2000, Changbai Mountain Academy of Sciences (CBMAS), Changbai Mountain Natural Reserve, began to carry out ex situ conservation of *R. sachalinensis*. More than 0.2 kg seeds and 60 seedings were transplanted into the ex situ conservation area in CBMAS. The ex situ conservation site is only decades of kilometers away from the main range of distribution of *R. sachalinensis*, and its main climate conditions are similar in a certain degree (Table 1). Up to 2007, about 500 individuals survived in ex situ conservation site. Based on our investigation, although there are morphological differences among natural populations of *R. sachalinensis*, we found distinct morphological differences between ex situ and natural populations (Figure 1, Table S1 and Figure S1).

**Table 1:** Comparison of climate and soil condition between native habitat and ex situ conservation site of *R. sachalinensis* populations.

Location	Altitude (m)	*T_mean_* (°C)	*T_min_* (°C)	*T_max_* (°C)	*P* (mm)	*Hr* (%)	Soil
**Tundra Zone**	2000	−3.4	−35. 9	29.6	1060. 6	73.0	Mountain tundra soil
**CBMAS**	763	3.6	−33.6	33.3	695.3	72.0	Albic dark-brownforest earths

*T_mean_*, annual average temperature; *T_min_*, extreme low temperature; *T_max_*, extreme high temperature; *P*, annual precipitation; *Hr,* relative humidity.

To understand these changes, we selected as study subjects *R. sachalinensis* populations i.e., three natural populations (TZ1, TZ2, TZ3) and one ex situ population (ESP), and detected the genetic, epigenetic, and HPLC fingerprint differentiation between them by using ISSR, MSAP and HPLC, with an intent to check whether ex situ population conserved representative natural populations, and to shed light on the strategy for ex situ conservation of *R. sachalinensis*.

## Materials and Methods

### Plant materials

We confirm that the permits were obtained from Changbai Mountain Natural Reserve where collecting took place. We confirm that the location accessed is not privately owned and all Sample collection complied with “Regulations of the People's Republic of China on Natural Reserves”.

Fresh leaves and roots of *R. sachalinensis* were collected in June, 2010 from three natural populations (Population TZ1, TZ2 and TZ3) and one ex situ population (ESP) cultivated in CBMAS (Table 2). ESP was reproduced by previous ex situ population in May, 2003, and they were almost at the same age. A random sample of 10–15 individuals was obtained from each population. Leaf samples were dried directly in silica gel and held at −80°C for DNA extraction. Root samples were air-dried under a shed and stored at −20°C until chemical extraction.

**Table 2:** Details of sampled *R. sachalinensis* populations.

Populations		Altitude (m)	Latitude (N)	Longitude (E)	Estimated population size	Number of samples		C
						Leaves	Roots	
***NP***	**TZ1**	2594	42°01′45.34″	128°03′56.87″	60	15	10	A_1∼15_
	**TZ2**	2293	42°02′40.20″	128°03′09.68″	70	15	10	B_1∼15_
	**TZ3**	2007	42°03′21.96″	128°03′55.29″	100	15	10	C_1∼15_
***ES*** **P**	**ESP**	763	42°22′53.28″	128°01′27.42″	320	15	10	D_1∼15_

*NP* is natural population of *R. sachalinensis*; *ES*P is ex situ population; C is code of individuals per populations.

### DNA extraction

Total DNA was extracted from silica-dried leaves using a Plant Genomic DNA kit (Dingguo, Beijing, China). DNA concentrations were quantified using a 752 UV-Vis spectrophotometer (Shanghai Spectrum Instruments, Shanghai, China). DNA quality was verified by electrophoresis on a 1.2% agarose gel. The DNA samples were diluted to 20 ng µl^−1^ and stored at −20°C until further analysis.

### ISSR

Thirty-six ISSR primers (Shenggong, Shanghai, China) designed by the University of British Columbia were screened initially using 12 individuals from the four populations. Twelve primers which produced clear and reproducible banding patterns were selected for amplification of all *R. sachalinensis* individuals in this study (Table 3). The optimized ISSR-PCR reaction system (25 µl) for *R. sachalinensis* was constructed of 2 µl template DNA, 2.5 µl of 10× *Taq* DNA polymerase buffer, 0.25 mM dNTPs, 1.5 mM MgCl_2_, 0.4 µM primer, 1.25 U of *Taq* polymerase (Takara, Dalian, China), and double-distilled water. The amplifications were carried out in a Mastercycler Gradient thermal cycler (Eppendorf, Hamburg, Germany) under the following conditions: pre-denaturation for 5 min at 94°C, followed by 40 cycles of denaturation for 30 s at 94°C, annealing for 60 s at various temperatures (see Table 3), and elongation for 90 s at 72°C, with a final extension for 10 min at 72°C.

**Table 3:** Sequences of ISSR primers and amplification results.

Primer	Sequence of primer (5′ to 3′)	*T_A_ (°C)*	*N_B_*	*N_PB_*	*PPB* (%)
**UBC807**	AGAGAGAGAGAGAGAGT	59.2	28	25	89.29
**UBC810**	GAGAGAGAGAGAGAGAT	51.6	34	26	76.47
**UBC825**	ACACACACACACACACT	51.6	31	28	90.32
**UBC840**	GAGAGAGAGAGAGAGAYC	59.2	31	25	80.65
**UBC841**	GAGAGAGAGAGAGAGAYT	52.7	37	30	81.08
**UBC842**	GAGAGAGAGAGAGAGAYG	58.1	28	19	67.86
**UBC856**	ACACACACACACACACYA	54.0	31	23	74.19
**UBC860**	TGTGTGTGTGTGTGTGRA	52.7	26	23	88.46
**UBC873**	GACAGACAGACAGACA	50.2	31	30	96.78
**UBC887**	DVDTCTCTCTCTCTCTC	54.0	27	20	74.07
**UBC888**	BDBCACACACACACACA	52.7	22	16	72.73
**UBC889**	DBDACACACACACACAC	55.4	26	20	76.92
**Total**			349	285	81.66

*B*: C, G or T; *D*: A, G or T; *Y*: C or T; *T_A_*: Annealing temperature; *N_B_*: Number of bands; *N_PB_*: Number of Polymorphic bands; *PPB:* Percentage of polymorphic bands.

ISSR-PCR products were separated via electrophoresis on a 1.8% agarose gel at 100 V for 150 min in 1×TBE (Tris base, boric acid, and 0.5 M EDTA) buffer, and stained with ethidium bromide (0.5 µg ml^−1^). Additionally, 100-bp ladders (Dingguo, Beijing, China) were loaded onto each gel. Images were identified using a gel-imaging system (Bio-Rad, CA, USA).

### MSAP

The methylation-sensitive amplification polymorphism (MSAP) technique, a modification of the amplified fragment length polymorphism (AFLP) method, exploits the differential sensitivity of a pair of isoschizomers (*Hpa* II and *Msp* I) to cytosine methylation. *Hpa* II and *Msp* I recognize and excise DNA sequences at CCGG restriction sites, but with different digestion sensitivities to cytosine methylation. *Msp* I cleaves DNA when no cytosines are methylated or when the internal C is methylated (5′-CmCGG-3′) on one or both DNA strands. *Hpa* II cleaves DNA in the case of no cytosine methylation or in the case of hemimethylation of external (5′-mCCGG-3′) or internal (5′-CmCGG-3′) cytosines [14]. The MSAP protocol and PCR amplification conditions were described by Schellenbaum et al. [15] with some modifications.

#### Digestion and ligation reactions

DNA samples were separately digested with *Eco*R I/*Hpa* II and *Eco*R I/*Msp* I (Fermentas, Shenzhen, China). Digestion and ligation were performed simultaneously in 20-µl volumes containing 200 ng DNA template, 5 U of T_4 _DNA ligase (Fermentas, Shenzhen, China), 5 U of *Eco*R I, 5 U of either *Msp* I or *Hpa* II, 5 pM of *Eco*R I adaptor (Eco-A1 and Eco-A2; Table 3), 50 pM of *Hpa* II-*Msp* I adaptor (HM-A1 and HM-A2; Table 3), 2.0 µl of 10× T_4 _DNA ligase buffer, and double-distilled water. The mixture was incubated at 37°C for 12 h, inactivated at 65°C, and stored at −20°C.

#### Pre-amplification PCR

The optimized Pre-amplification PCR reaction system (20 µl) for *R. sachalinensis* was constructed of 2 µl digestion-ligation products, 2.5 pM of each pre-amplification primer (E00 and HM00; Table 4), 1 U of *Taq* polymerase, 2.0 µl of 10× *Taq* DNA polymerase buffer, 0.20 mM dNTPs, 1.5 mM MgCl_2_, and double-distilled water. The pre-amplification conditions consisted of pre-denaturation for 5 min at 94°C, followed by 30 cycles of denaturation for 30 s at 94°C, annealing for 60 s at 56°C, and elongation for 80 s at 72°C, with a final extension for 10 min at 72°C and storage at −20°C.

**Table 4:** Adaptors and primers used for MSAP analysis.

Adaptors	
Eco-A1 5′- CTCGTAGACTGCGTACC-3′ Eco-A2 5′- AATTGGTACGCAGTC -3′	HM-A1 5′- GATCATGAGTCCTGCT -3′ HM-A2 5′- CGAGCAGGACTCATGA -3′
Preamplification primers	
E00 5′- GACTGCGTACCAATTCA -3′	HM00 5′- ATCATGAGTCCTGCTCGG -3′
**Selective amplification primers**	
*Eco*R primer	*Msp*I/*Hpa*II primer
E7 = 5′- GACTGCGTACCAATTCAAG -3′	HM13 = 5′- ATCATGAGTCCTGCTCGGTCG -3′
E7 = 5′- GACTGCGTACCAATTCAAG -3′	HM14 = 5′- ATCATGAGTCCTGCTCGGTCC -3′
E7 = 5′- GACTGCGTACCAATTCAAG -3′	HM15 = 5′- ATCATGAGTCCTGCTCGGTTG -3′
E7 = 5′- GACTGCGTACCAATTCAAG -3′	HM16 = 5′- ATCATGAGTCCTGCTCGGTTA -3′
E7 = 5′- GACTGCGTACCAATTCAAG -3′	HM17 = 5′- ATCATGAGTCCTGCTCGGTGA -3′
E7 = 5′- GACTGCGTACCAATTCAAG -3′	HM18 = 5′- ATCATGAGTCCTGCTCGGTAC 3′

#### Selective amplification PCR

Pre-amplification products were diluted 1 to 20 (v/v) with ddH_2_O and selective amplification using six different primer combinations (Table 4) The optimized selective amplification PCR reaction system (25 µl) for *R. sachalinensis* was constructed of 2 µl diluted pre-amplification product, 0.2 mM dNTPs, 2 pM *Eco*R I and *Msp* I/*Hpa* II primers, 1 U of *Taq* polymerase, 2.5 µl of 10× *Taq* DNA polymerase buffer, 0.20 mM dNTPs, 1.5 mM MgCl_2_, and double-distilled water. For selective amplification, touchdown PCR conditions were as follows: pre-denaturation for 5 min at 94°C, followed by 30 cycles of 30 s at 94°C, 30 s at 65°C decreasing by 0.7°C per cycle, and 80 s at 72°C, then followed by 23 cycles of 30 s at 94°C, 30 s at 55°C, and 80 s at 72°C, and a final extension step of 10 min at 72°C.

After selective amplification, the products were mixed 1∶1 with a loading buffer (98% deionized formamide, 10 mM EDTA, 0.1% bromophenol blue, and 0.1% xylene cyanol), denatured at 95°C for 5 min, and separated on 6% denaturing polyacrylamide gels (6% polyacrylamide and 7 M urea) in 1× TBE buffer at 70 W for 4.5 h. Gels were stained according to the silver staining method [16].

### HPLC fingerprint analysis

HPLC fingerprint, a comprehensive and quantifiable identification method, is able to reveal chemical information about herbal medicines using chromatograms, spectrograms, and other graphs derived by analytical and chemical techniques [17], [18]. The State Food and Drug Administration of China (SFDA) require that all injections made from herbal medicines or their raw materials must be standardized by HPLC fingerprint [19].

#### Standards and solvents

Salidroside used as standards, were provided by Jilin Food and Drug Administration. Methanol used as solvents, were of HPLC grade and were purchased from Fisher Scientific (USA). After intensive drying salidroside was weighed and dissolved in 1 mL of methanol to achieve serial concentrations, and three injections were performed for each dilution. The standard curve was calibrated using the linear least-squares regression equation derived from the peak area. The concentration of salidroside in different samples was calculated according to the regression parameters derived from the standard curves.

#### Extraction of chemical composition

To extract chemical composition, root samples from the four populations were pulverized, and the powder was then screened through 180-µm sieves. Fine powder (200 mg) was accurately weighed; 10 ml of 50% methanol was then added and the mixture was weighed again. The mixture was extracted by ultrasonication for 1 h at 60°C. After cooling, 50% methanol was added to restore the initial weight. The supernatant fluid was filtered through a 0.45-µm membrane filter, and the filtrate was analyzed by HPLC.

#### HPLC analysis

Chromatographic separations were performed on an Agilent 1100 HPLC system (Agilent, Böblingen, Germany) equipped with a quaternary pump, autosampler, degasser, automatic thermostatic column compartment, DAD detector, and a computer with Chemstation software for analysis of the HPLC data. An Agilent C_18_ reversed-phase column (250×4.6 mm; 5 µm) was used with the column temperature set at 25°C. The mobile phase consisted of 15∶85 (v/v) methanol-ddH_2_O, which was maintained for 40 min. The flow rate was 1.0 ml min^−1^ and the injection volume was 20 µl. The detection wavelength was set to 277 nm.

### Data analysis

#### ISSR fragment analysis

ISSR-amplified DNA fragments (bands) were scored as present (1) or absent (0), and a data matrix of the ISSR banding patterns of all populations was assembled for further analysis. The software program POPGENE v1.31 [20] was used to estimate the following genetic diversity parameters: Shannon’s information index (*I*) [21], percentage of polymorphic loci (*PPL*), and genetic distance. Total genetic diversity (*Ht*) and mean genetic diversity within populations (*Hs*) were tested using Nei’s [22] genetic diversity statistics. The proportion of diversity among populations was calculated as the coefficient of gene differentiation, *G_ST_*, according to the equation *G_ST_* = (*Ht−Hs*)*/Ht*. Gene flow was estimated using the formula: *Nm = 0.5×*[(*1−G_ST_*)*/G_ST_*] [23]. These calculations were carried out using POPGENE v1.31. A hierarchical analysis of molecular variance (AMOVA) was employed to examine population genetic differentiation (*F_ST_*) within and among the four different altitudinal populations using AMOVAPREP and AMOVA 1.55 [24]. The significance of this *F_ST_* was tested with 1,000 random permutations. A UPGMA tree was constructed based on genetic distance [25] among different populations to determine their genetic relationships using NTsys-pc v2.02a [26].

#### MSAP fragment analysis

Only clear fragment patterns were considered. *Hpa* II and *Msp* I have different sensitivities to methylation patterns: *Hpa* II is only sensitive to external hemimethylated cytosine (single-strand methylation), whereas *Msp* I only recognizes internal fully methylated cytosine (methylation of both strands). We therefore scored the differential methylation pattern based on band presence or absence [27]. MSAP banding patterns were classified into four types: Type 1/1 bands represented non-methylated loci (bands present in both profiles), Type 1/0 bands corresponded to loci hemi-methylated at the external C of the restriction site (bands present in *Eco*R I/*Hpa* II only), Type 0/1 bands indicated full methylation of the internal cytosine of both strands (bands present in *Eco*R I/*Msp* I only), and Type 0/0 bands represented non-informative loci (bands absent in both profiles). Non-informative loci were removed from our dataset because they could not discriminate between hyper-methylation and nucleotide mutation, and the number of hemi-methylated and internal fully methylated loci was then counted in each sample. Ratios of hemi-methylation, internal full methylation, and total methylation (Type 1/0+Type 0/1) were calculated against the total number of detected bands (Type 1/1+ Type 1/0+ Type 0/1). To estimate population epigenetic structure in the four different habitats, MSAP data were also converted into a methylation-susceptible matrix according to Herrera and Bazaga [28]. More specifically, band absence in either *Eco*R I/*Msp* I or *Eco*R I/*Hpa* II profiles was scored as “1” and band presence in both *Eco*R I/*Msp* I and *Eco*R I/*Hpa* II profiles was scored as “0”; bands absent in both *Eco*R I/*Msp* I and *Eco*R I/*Hpa* II profiles were treated as “missing”, as they could have been produced by either genetic or epigenetic factors. The resulting data were analyzed with POPGENE v1.31 [20] to calculate diversity parameters, including genetic differentiation (*G_ST_*), Nei’s gene diversity, gene flow (*Nm*), and genetic distance, to search for epigenetic differentiation. AMOVAPREP and AMOVA 1.55 [24] were used to further examine population epigenetic differentiation within and among the four different altitudinal populations. The software package Ntsys-pc v2.02a [26] was used for Mantel testing and UPGMA dendrogram generation.

#### HPLC fingerprint analysis

HPLC fingerprints of *R. sachalinensis* root extracts were analyzed using the professional software package Similarity Evaluation System for Chromatographic Fingerprint of Traditional Chinese Medicine (2004 version), recommended by the SFDA of China for evaluating similarities of traditional Chinese medicine chromatographic profiles [29]. Hierarchical cluster analysis of samples from the four different altitudinal populations was performed based on retention time and peak area variation patterns using the UPGMA method as implemented in Ntsys-pc v2.02a [26].

#### Correlation analysis

We used the DPS 7.05 data processing system (Zhejiang University, Hangzhou, China) to analyze relationships among environment, heredity, and HPLC fingerprint in different populations of *R. sachalinensis.*

## Results

### Genetic polymorphism and population structure

To assess the genetic diversity of *R. sachalinensis*, we surveyed 12 ISSR primers in 60 individuals from the different populations sampled in 2010. A total of 349 bands (Table 3) were produced, which corresponded to an average of 29.08 bands per primer. Of these bands, 81.66% (Table 3) were polymorphic among the 60 individuals.

#### Genetic diversity and differentiation

At the species level, the percentage of polymorphic loci (*PPL*) and Shannon’s information index (*I*) were 97.71% and 0.4299, respectively. At the population level, genetic diversity of *R. sachalinensis*, the ESP, was relatively high, while TZ1, TZ2, and TZ3 genetic diversities were more or less similar to one another. *PPL* ranged from 63.90% to 50.14%, with an average of 55.37%. *I* ranged from 0.3195 to 0.2351, with an average of 0.2743 (Table 5).

**Table 5:** Genetic diversity of *R. sachalinensis* distributed among different altitudinal populations.

Populations	*PPL* (%)	*I*	*G_ST_*	*Nm*
**TZ1**	52.15	0.2633		
**TZ2**	50.14	0.2351		
**TZ3**	55.30	0.2794		
**ESP**	63.90	0.3195		
**Mean**	55.37	0.2743		
**Species level**	97.71	0.4299	0.3490	0.9327

*PPL*: percentage of polymorphic loci; *I:* Shannon’s information index; *G_ST_*: genetic differentiation coefficient; *Nm*: gene flow.

The calculated coefficient of genetic differentiation (*G_ST_*) suggested that genetic differentiation of *R. sachalinensis* populations was relatively high (0.3490), indicating that about 34.90% of the genetic variation was among populations. The calculated gene flow was 0.9327 migrants (*Nm*) per generation (Table 5). AMOVA of the four populations revealed that 45.12% and 54.88% of the observed genetic variation occurred among and within populations, respectively (Table 6). The AMOVA results were consistent with Nei’s genetic structure estimations (Table 6). Based on ISSR data, the mean overall *F_ST_* value for individual loci was 0.451. This value was significantly different from zero (*P*<0.001).

**Table 6:** Genetic variation within and among populations of *R. sachalinensis* revealed by AMOVA.

Source of variation	df	SSD	MSD	Variancecomponent	%Total	*F_st_*	*P* value
**Among populations**	3	1410.1833	470.061	28.9868	45.12	0.451	<0.0010
**Within populations**	56	1974.5333	35.260	35.2595	54.88		
**Total**	59	3384.7167	505.321	64.2463	100		

*df* the degree of freedom, *SSD* sums of the squared deviations, *MSD* mean squared deviations, *%Total* the percentage of the total variance.

#### Cluster analysis

**Figure 2:**
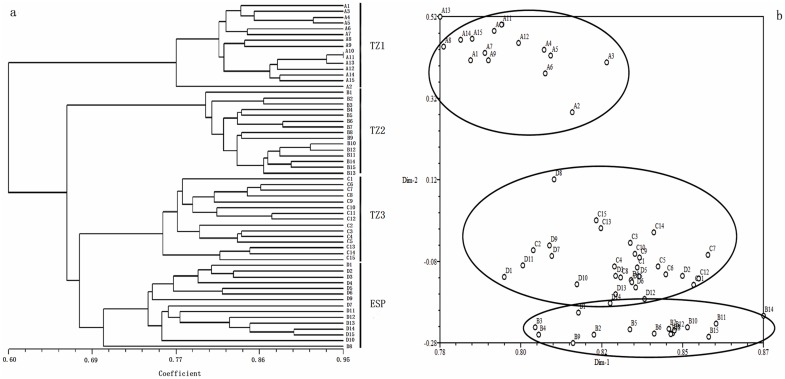
UPGMA dendrogram (a) and principal coordinate plot (b) based on ISSR markers for *R. sachalinensis* individuals from different populations. Black dots represent sampled individuals (for individual codes, see Table 2).

Based on the ISSR markers data, a UPGMA dendrogram of all sampled *R. sachalinensis* individuals was reconstructed using the Nei genetic distance matrix. In this dendrogram, all the individuals in a given population formed a distinct cluster. Each population had its own node. The 60 *R. sachalinensis* individuals from natural and ex situ conservation populations clustered into three major groups, with TZ1 and TZ2 comprising single branches and TZ3 and ESP clustered together on a third branch (Figure 2a).

A principal coordinates (PCO) analysis based on genetic distances provided a spatial representation of the 60 individuals from the four sampled populations (Figure 2b). The two-dimensional PCO plot clearly differentiated populations TZ1 and TZ2. Individuals from TZ3 and ESP clustered together near the bottom of the PCO plot. There were several individuals with minimal overlap between TZ3 and ESP.

### DNA Methylation Levels

**Figure 3:**
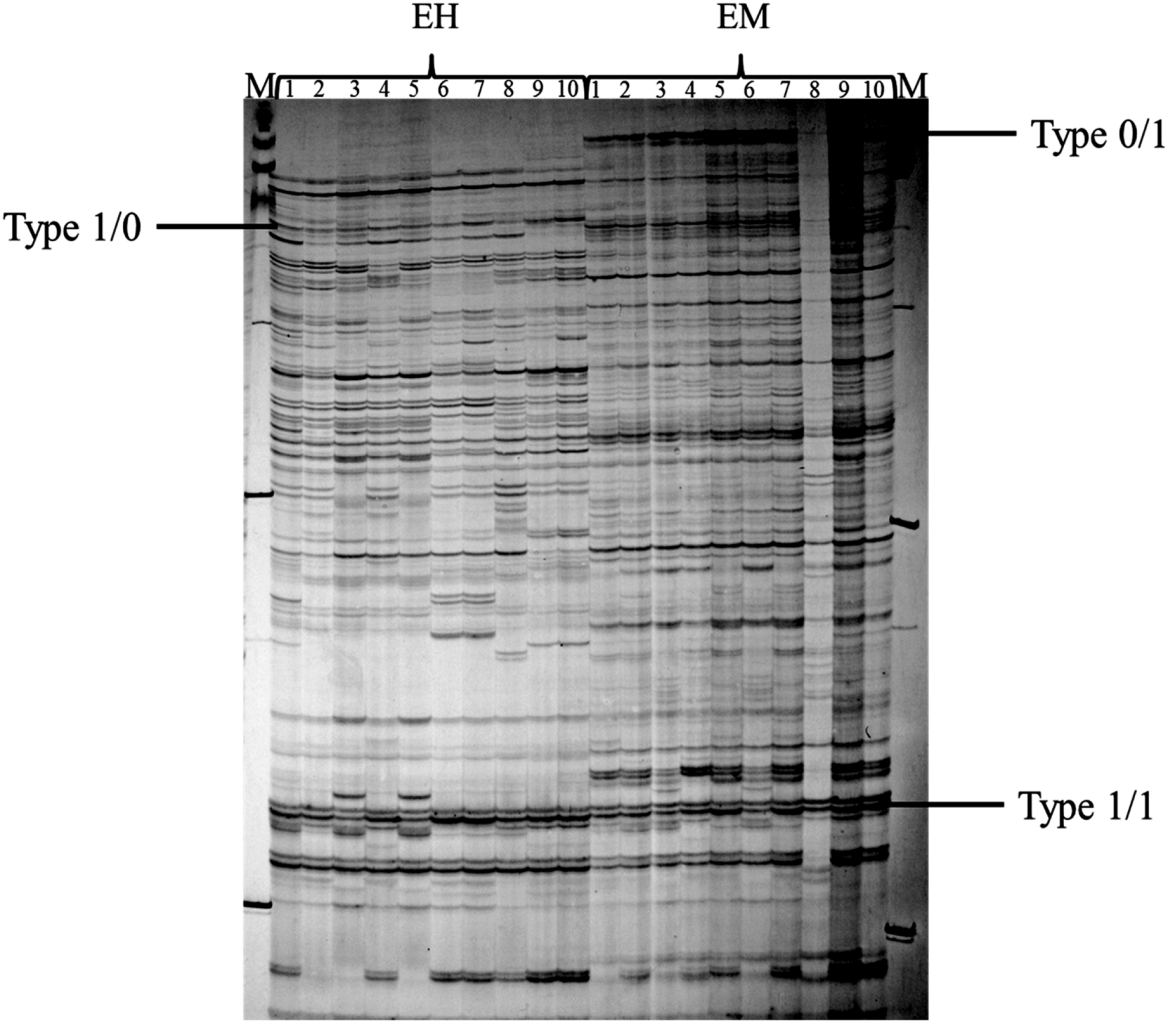
DNA methylation patterns in different populations of *R. sachalinensis.* * EH* and *EM* represent *Eco*R I/*Hpa* II and *Eco*R I/*Msp* I digestion combinations, respectively. Type 1/1 bands were present in both *Eco*R I/*Hpa* II and *Eco*R I/*Msp* I digestion combinations. Type 1/0 bands were present only in *Eco*R I/*Hpa* II combinations, and Type 0/1 bands were present only in *Eco*R I/*Msp* I digestions.

DNA methylation levels in the different *R. sachalinensis* populations were evaluated by MSAP fingerprint. Based on band absence or presence, cytosine methylation patterns were classified into four types: Type 1/1, Type 1/0, Type 0/1, and Type 0/0 (Figure 3; Table 7). After removing Type 0/0 bands, which corresponded to non-informative loci, total methylation levels in populations TZ1, TZ2, TZ3, and ESP4 were 38.18%, 34.80%, 30.62%, and 25.00%, respectively; corresponding internal full methylation levels were 20.83%, 19.79%, 18.75%, and 15.17%, and hemi-methylation levels were 17.35%, 15.01%, 11.87%, and 9.83%, respectively (Table 7). The four *R. sachalinensis* populations thus differed to some degree with respect to DNA methylation levels. Total methylation, internal full methylation, and hemi-methylation levels gradually decreased along the altitudinal gradient, with TZ1 the most methylated and ESP the least.

**Table 7:** DNA methylation levels of four different populations of *R. sachalinensis*.

Types	Methylation Status	Populations			
		TZ1	TZ2	TZ3	ESP
**Type 1/1**	CCGG CCGG GGCC GGCC	531	547	545	603
**Type 1/0**	CCGG GGCC	149	126	95	79
**Type 0/1**	CCGG GGCC	179	166	150	122
**Total bands**	–	859	839	800	804
**Hemi-methylation ratio (%)**	–	17.35	15.01	11.87	9.83
**Internal full methylation Ratio (%)**	–	20.83	19.79	18.75	15.17
**Total methylation (%)**	–	38.18	34.80	30.62	25.00

Total no. of bands of three types = I+II+III, ratio for hemi-methylation of external cytosine (%) = II/(I+II+III)×100%, ratio for full methylation of internal cytosine (%) = III/(I+II+III)×100%, ratio for total methylation (%) = (II+III)/(I+II+III). Each of populations contains with 10 individuals.

### Epigenetic polymorphism and population structure

To estimate population epigenetic structure, MSAP data were converted into a methylation-susceptible matrix according to Herrera and Bazaga [28]. We then used POPGENE, AMOVA, and Ntsys-pc software to calculate epigenetic polymorphism levels and population structure of the four populations.

#### Epigenetic diversity and differentiation

At the species level, *PPL* and *I* were 85.99% and 0.3778, respectively. At the population level, epigenetic diversity of ESP was relatively high, while epigenetic diversity of TZ1, TZ2 and TZ3 gradually decreased in that order. *PPL* ranged from 53.85% to 21.39%, with an average of 34.91%. *I* ranged from 0.2443 to 0.0971, with an average of 0.1563 (Table 8).

**Table 8:** Epigenetic diversity of *R. sachalinensis* distributed in different altitudinal populations.

Populations	*PPL* (%)	*I*	*G_ST_*	*Nm*
**TZ1**	53.85	0.2443		
**TZ2**	39.09	0.1757		
**TZ3**	25.29	0.1080		
**ESP**	21.39	0.0971		
**Mean**	34.91	0.1563		
**Species level**	85.99	0.3778	0.5892	0.3486

The coefficient of genetic differentiation (*G_ST_* = 0.5892) indicated that a certain degree of epigenetic differentiation existed among the four *R. sachalinensis* populations, corresponding to about 58.92% of the observed epigenetic variation. The calculated gene flow was 0.3486 migrants (*Nm*) per generation (Table 8). AMOVA of the four populations revealed that 63.87% of the epigenetic variation occurred among populations, while 36.13% occurred within populations (Table 9). The AMOVA results were consistent with values calculated based on Nei’s genetic structure (Table 9). Based on MSAP markers, mean overall *F_ST_* for individual loci was 0.639, which was significantly different from zero (*P*<0.001).

**Table 9:** Epigenetic variation within and among populations of *R. sachalinensis* revealed by AMOVA.

Source of variation	df	SSD	MSD	Variance component	%Total	*F_st_*	*P* value
**Among populations**	3	3543.4750	1181.158	111.7928	63.87	0.639	<0.0010
**Within populations**	36	2276.3000	63.2310	63.2305	36.13		
**Total**	39	5819.7750	1244.389	175.0233	100		

#### Cluster analysis

**Figure 4:**
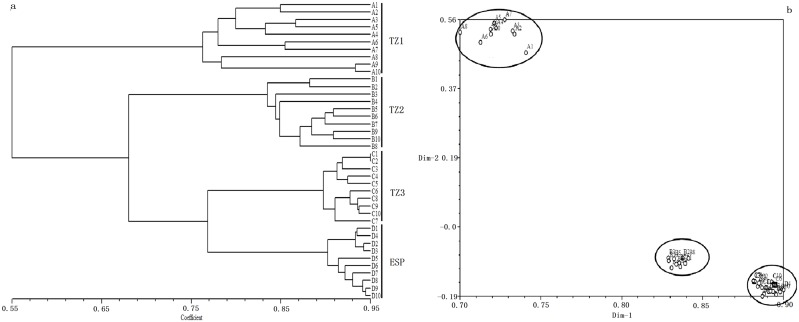
UPGMA dendrogram (a) and principal coordinate plot (b) based on MSAP markers for *R. sachalinensis* distributed in different altitudinal populations. Black dots represent sampled individuals (for individual codes, see Table 2).

Based on the methylation-susceptible matrix, a UPGMA dendrogram was reconstructed from epigenetic distances for 40 *R. sachalinensis* individuals from the different altitudes. This dendrogram was similar to the ISSR marker-based dendrogram, with individuals clustering according to population into three distinct groups. In the dendrogram, TZ1 and TZ2 comprised two groups, and TZ3 and ESP were joined together along a third major branch (Figure 4a).

PCO analysis based on epigenetic distances among the 40 sampled individuals was used to obtain a spatial representation of their relationships (Figure 4b). The two-dimensional PCO plot clearly differentiated TZ1 and TZ2. TZ3 and ESP populations were clustered near the top of the PCO plot; their epigenetic distances were very small, but no individual was shared between them.

### Chemical fingerprint

#### Salidroside content

The salidroside content in different populations significantly varied, from 0.25 to 11.12 mg/g, as listed in Table 10. The salidroside content of natural populations (TZ1, TZ2 and TZ3) were 1.54, 11.12, 6.96 mg/g respectively. The ex situ population (ESP) was the lowest, only 0.25 mg/g.

**Table 10:** Comparison of salidroside content among *R. sachalinensis* populations.

Populations	Standard equation	*R^2^*	Salidroside content (mg/g)
**TZ1**	*y = *2401.4*x–*11.567	0.9998	1.54
**TZ2**			11.12
**TZ3**			6.96
**ESP**			0.25

#### Similarity of HPLC fingerprint

**Figure 5:**
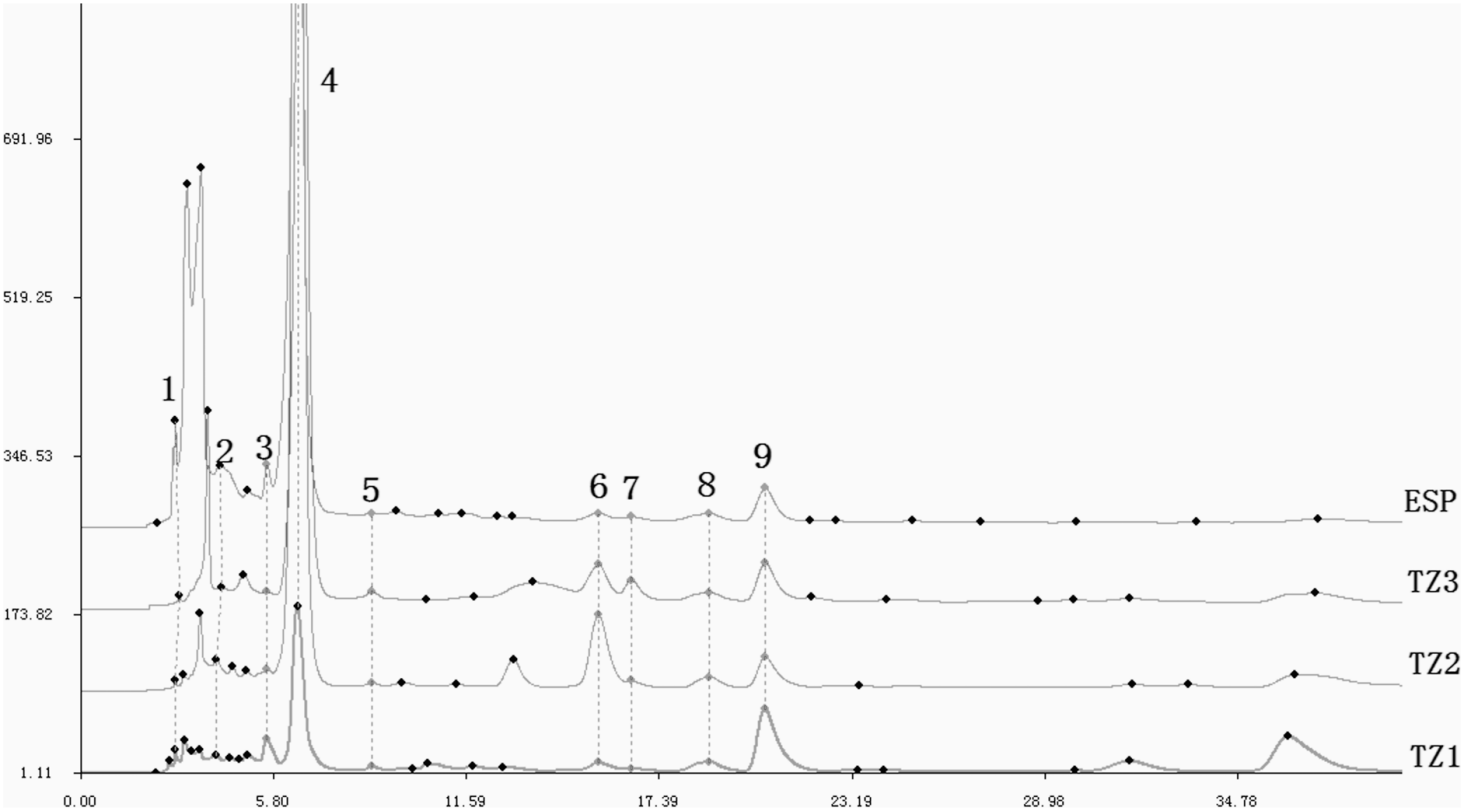
HPLC fingerprints of *R. sachalinensis* populations. HPLC fingerprints obtained from natural populations (TZ1, TZ2 and TZ3) and ex situ conservation population (ESP). Black dots represent peak. 1–9 correspond to 9 common peaks.

The major HPLC fingerprint of *R. sachalinensis*, were obtained by HPLC. HPLC fingerprints of *R. sachalinensis* from the different populations revealed an abundant diversity of HPLC fingerprint. Twenty chemical components were identified based on peak retention times, with relative peak areas corresponding to concentration (Figure 5). HPLC fingerprints were compared using the Similarity Evaluation System for Chromatographic Fingerprint of Traditional Chinese Medicine (2004 version) (Table 11). Based on this analysis, the higher similarity of HPLC fingerprint, over 0.89, was found among natural populations (TZ1, TZ2 and TZ3). While the similarities between ESP and natural populations were less than 0.85.

**Table 11:** Similarities of chromatographic fingerprints of *R. sachalinensis* distributed in different altitudinal populations.

Populations	TZ1	TZ2	TZ3	ESP
**TZ1**	1	0.895	0.899	0.762
**TZ2**	0.895	1	0.992	0.781
**TZ3**	0.899	0.992	1	0.843
**ESP**	0.762	0.781	0.843	1

**Figure 6:**
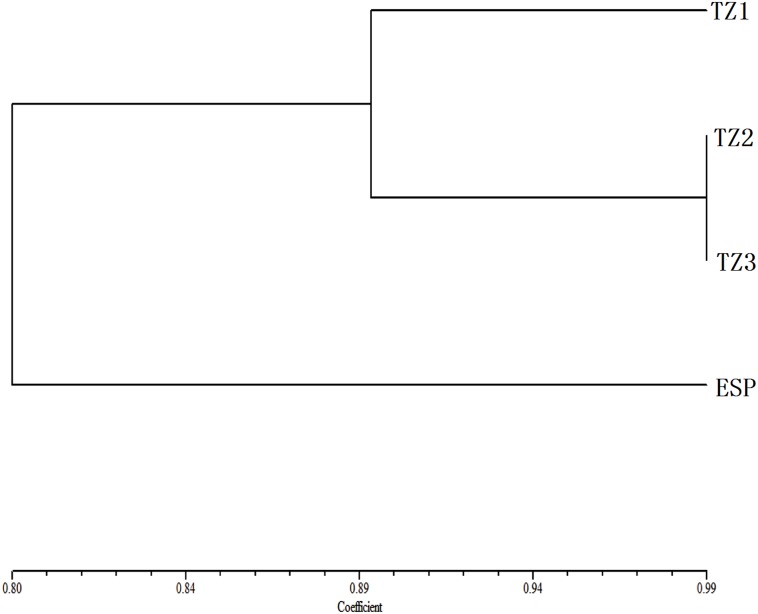
UPGMA dendrogram analysis derived from the Chromatographic fingerprints of *R. sachalinensis* distributed in different altitudinal populations.

We then performed cluster analysis on *R. sachalinensis* populations using Ntsys-pc based on HPLC fingerprint similarities (Figure 6). This analysis uncovered three major clusters: TZ1, ESP, and a cluster consisting of TZ2 and TZ3. These results are obviously different from those obtained from cluster analysis of genetic and epigenetic marker data.

### Correlation analysis

Correlation analysis of altitude, genetic distance, epigenetic distance, DNA methylation level, and HPLC fingerprint similarities generated interesting results (Table 12). Genetic and epigenetic distances were significantly correlated (*R^2^* = 0.98). DNA methylation level and altitude were also significantly correlated with one another (*R^2^* = 0.90), and negatively correlated with HPLC fingerprint similarity (*R^2^* = −0.88). Chemical fingerprint similarity and altitude were apparently negatively correlated (*R^2^* = −0.95) as well. Genetic and epigenetic distances were somewhat correlated with HPLC fingerprint similarity, but the correlation coefficients were not significant (*R^2^* = 0.35 and *R^2^* = 0.48).

**Table 12:** Correlations between genetic structure, epigenetic structure, altitude, and chromatographic fingerprints.

Correlation coefficientand *P*-value	Altitude	Genetic distance	Epigeneticdistance	DNA methylationlevel	Similarity ofchromatographicfingerprints
**Altitude**	1	−0.17, *p* = 0.7405	−0.35, *p* = 0.4441	0.90**, *p* = 0.0054	−0.95**, *p* = 0.0012
**Genetic distance**	−0.17, *p* = 0.7405	1	0.98**, *p* = 0.0002	−0.44, *p* = 0.3186	0.35, *p* = 0.4380
**Epigenetic distance**	−0.35, *p* = 0.4441	0.98**, *p* = 0.0002	1	−0.61, *p* = 0.1441	0.48, *p* = 0.2758
**DNA methylation level**	0.90**, *p* = 0.0054	−0.44, *p* = 0.3186	−0.61, *p* = 0.1441	1	−0.88**, *p* = 0.0099
**Similarity of** **chromatographic** **fingerprints**	−0.95**, *p* = 0.0012	0.35, *p* = 0.4922	0.48, *p* = 0.2758	−0.88**, *p* = 0.0099	1

*p<0.05 **p<0.01.

## Discussion

### Population genetic structure

ISSR revealed a high of genetic diversity in endangered *R. sachalinensis*, with average of 2.53% percentage of polymorphic loci (*PPL*) and 0.2593 Shannon’s information index (*I*) in three natural populations. An allozyme analysis of *R. sachalinensis* natural population genetic structure by Yan et al. [30] found a low level of genetic variation. Two factors may be responsible for these conflicting results and our observations. First, allozymes reflect only a subset of genes, i.e., those that are expressed, rather than the majority of genes including non-functional ones, whereas DNA molecular markers can more widely sample the genome. Second, breeding system, life form, and natural selection play very important roles in genetic differentiation. Hamrick and Godt [31] listed eight factors that affect genetic variation. In particular, breeding system and gene flow have particularly strong impact on genetic structure, with widespread species with high levels of gene flow and outcrossing having higher genetic variation. Generally, long-lived perennial species with a mixed breeding system have relatively high genetic diversity [32]. *R. sachalinensis* is a perennial alpine herb with predominant outcrossing, characteristics associated with high genetic diversity. In addition, *R. sachalinensis* reproduces both sexually and clonally via sprouting rhizomes. Although “pollen limitation” was invoked by Yan et al. [30] to explain the low levels of genetic variation they observed in *R. sachalinensis* populations, recent studies have demonstrated that bumblebees play a positive role in pollen dispersal of alpine plants [33], [34]. These characteristics, i.e., a dual reproductive system and pollen dispersal, can be viewed as a strategy for maximal heterozygosity and reproductive success, and are common in alpine plants exposed to such drastic environments [35], [36].

Ex situ population (ESP) had higher genetic diversity (*PPB* = 63.9% and *I* = 0.3195) than the other three natural populations. Most researches show that the populations of ex situ populations have a low level of genetic diversity, but we found a different result. On the one hand, rich genotypes of species and cultivation conditions may be the major reason. More than 0.2 kg seeds and 60 seedings from natural populations were transplanted into the ex situ conservation area in CBMAS. The quantity of *R. sachalinensis* ensured variation of genotypes and genetic information, and cultivation conditions are good at establishing artificial population of *R. sachalinensis*; On the other hand, according to archived cultivation information, the artificial population of *R. sachalinensis* is derived from natural populations growing at 2,000 m, and has experienced reproduction by seed (sexual reproduction) for several generations. All these factors are better for holding a higher level of genetic diversity.

Analysis of ISSR marker data using different approaches (AMOVA and Nei’s genetic diversity) found similar results. Partitioning of genetic divergence by AMOVA revealed that most of the genetic variation, 54.88%, was within-population variation. The observed level and pattern of genetic differentiation is consistent with the reproductive strategy of *R. sachalinensis*, a gravity-dispersed species with mixed mating.

High genetic differentiation (*G_ST_* = 0.3490) was observed between natural *R. sachalinensis* populations and ex situ population. An effective gene flow of more than four migrants per generation is generally sufficient to counteract genetic drift and to prevent genetic differentiation between populations [37]. In this study, a gene flow of 0.9327 was estimated, indicating that some gene exchange is occurring among *R. sachalinensis* populations. However, this relatively low gene flow was not the only contributor to the high genetic differentiation observed for *R. sachalinensis* [38].

### Population epigenetic structure

Genetic variation has traditionally been regarded as the fundamental source of adaptation capacity of species to respond to environmental stress [39], [40]. However, in recent years, epigenetic variation has also been linked to species adaptive flexibility [41], [42], [43], [44], [45], [46], [47].

At the species level, high levels of epigenetic variation were uncovered in *R. sachalinensis* from different populations, with PPB = 85.99%, *I* = 0.3778, and *G_ST_* = 0.5892 (Table 5). Most of the epigenetic variation was among population, as evidenced by a value of 63.87% revealed by AMOVA (Table 6). Because gene flow was relatively low (*Nm* = 0.3486), it may not be the major factor responsible for the high epigenetic differentiation of *R. sachalinensis*. During the past decade, many studies have demonstrated that epigenetic variation is usually associated with phenotypic novelties that can also contribute to the ability of plants to respond to diverse environments [28], [47], [48], [49]. Because populations of *R. sachalinensis* distributed at different altitudes differ greatly in morphology, environmental conditions at different altitudes may be a major factor responsible for the observed high epigenetic variation. Additionally, *R. sachalinensis* DNA methylation levels, including total methylation, internal full methylation, and hemi-methylation levels, gradually decreased along an altitudinal gradient. TZ1, at the highest altitude, was the most methylated, whereas ESP as an ex situ population, at the lowest altitude, was the least. Previous studies of epigenetic variation in *Pisum sativum* L., *Nicotiana tabacum* L., and *Zea mays* L. illustrate that species can adapt to adverse environmental conditions by altering DNA methylation levels [50], [51], [52]. Consequently, epigenetic variation in *R. sachalinensis* was a response to environmental stress. Although these epigenetic variation are favor for survival of *R. sachalinensis* in ex situ conservation, a long-term view there is adversely for maintaining the original property of *R. sachalinensis*.

### HPLC fingerprint of *R. sachalinensis*

Plant metabolites have appeared over the course of evolution as plants adapted to their environments, and play important roles in many processes, such as physiology regulation and environmental response [53]. In additional, *R. sachalinensis* is a kind of medicinal plant, so HPLC fingerprint is self-evident importance of maintaining medicinal properties. In this study, we surveyed chromatographic fingerprints of *R. sachalinensis* individuals from different populations using HPLC fingerprint (Table 11). According to our results, there were notable differences in HPLC fingerprints among the different populations; ESP was the most distinct, as corroborated by the results of cluster analysis, and the content of salidroside is the lowest, only 0.25 mg/g. (Figure 6 and Table 10). These results are clearly different from those obtained from cluster analysis of genetic and epigenetic markers. These noticeable differences may be explained by the fact that ESP, as an ex situ population at 763 m, has experienced an environment unlike the alpine conditions of the other populations.

It is well known that HPLC fingerprints are influenced by heredity and environmental factors. For *R. sachalinensis* individuals distributed in different populations, environmental factors associated with their habitats are the major influences on HPLC fingerprint. Previous studies of HPLC fingerprint in *R. sachalinensis* have demonstrated that environmental factors such as temperature, light quality, soil, and precipitation affect secondary metabolite formation and accumulation [54], [55]. Alpine environmental stress may thus be responsible for the observed differences in HPLC fingerprints among the different populations. Because ESP, at 763 m, has been subjected to the most divergent environmental stress compared with natural populations, its HPLC fingerprint would be more obviously different. These changes to the original property preservation of *R. sachalinensis* may be adversely.

### Correlations among altitude, genetic and epigenetic structure, and HPLC fingerprint

Plant genetic structure, epigenetic structure, and chromatographic fingerprints are all affected by environmental factors. We performed a correlation analysis to gain a better understanding of their inter-relationships, and uncovered close relationships among these factors.

According to our results, genetic structure was significantly correlated with epigenetic structure (*R^2^* = 0.98), consistent with the similar dendrograms generated by cluster analysis of genetic and epigenetic marker data. A correlation between genetic and epigenetic changes has been previously suggested, but an equivalent relationship as revealed in this study has seldom been experimentally demonstrated [11]. Our results indicate that the pattern of epigenetic variation (cytosine methylation) in *R. sachalinensis* from different altitudes may be correlated with genomic sequence variation (genetic variation), and that epigenetic structure may be influenced by genetic structure.

Another characteristic of the epigenetic variation observed in *R. sachalinensis* was that it was correlated positively with altitude (*R^2^* = 0.90) and that HPLC fingerprint similarity and altitude were negatively correlated (*R^2^* = −0.95). Altitudinal gradients are associated with substantial changes in various environmental conditions over a short distance, while epigenetic variation and HPLC fingerprints are affected by environmental factors [56], [57]. Changes in epigenetic variation and HPLC fingerprint are thus inevitable along an altitudinal gradient.

There is an interesting result that epigenetic variation and HPLC fingerprints were negatively correlated (*R^2^* = −0.88). It is well known that plant metabolism and DNA methylation have strong organ and tissue specificity. So the correlate methylation patterns of leaves with HPLC fingerprint variation of roots may be unreasonable. However, this correlation may exist, because as a very important tissue of photosynthesis, DNA methylation of leaves may be an indirect influence on the formation and accumulation of secondary metabolites of roots. It may be a point of interest in the future.

Through correlation analysis of altitude, genetic structure, epigenetic structure, and HPLC fingerprint patterns, we determined that environmental conditions are a major factor influencing genetic and epigenetic structure and HPLC fingerprint. The clear genetic variation among *R. sachalinensis* populations is the result of a long process of adaptation to alpine environmental stress [58]. Environmental factors can also more directly regulate epigenetic variation to produce adaptive changes; when the environment changes, DNA methylation can regulate gene expression to allow rapid response to environmental fluctuation [28], [59], [60]. Recent studies of natural populations have indicated that alterations in cytosine methylation status are usually associated with changes in physiological and morphological traits of individuals, and that these phenotypic novelties are available for the action of natural section [28], [48]. In this study, we also uncovered alterations in *R. sachalinensis* phenotypes among the different altitudinal populations, including changes in morphology and HPLC fingerprint.

Taken together, for natural populations of *R. sachalinensis*, although they have a higher level of genetic, their gene flow is lower, only 0.9327. So besides ex situ conservation, the most important way to conserve *R. sachalinensis* is through the protection of the habitat and reintroduction in which it lives by in situ conservation. Furthermore, for ex situ conservation and artificial cultivation of endangered plants, especially medicinal plants, not only should their survival be considered, but also the impact of environmental factors on their genetic and epigenetic structures and physiological metabolism characteristics.

## Conclusions

The results of this study demonstrate that environmental conditions in different habitats are major factor influenced on hereditary characteristic of species. The ex situ population of *R. sachalinensis* have been generated some variations especially epigenetics and physiological metabolism in a short period (for nearly 10 years until June, 2010). Those mean that it is a certain degree of difficult to representative natural populations from the ex situ conservation population.

In addition, *R. sachalinensis* is an important, endangered medicinal plant species. Combined knowledge of HPLC fingerprint in exploited medicinal plants should provide a useful guide for their efficient utilization, breeding, and management.

## Supporting Information

Figure S1 **Morphological variation displayed by** ***R. sachalinensis*** **plants from different altitudes on Changbai Mountain.** (a) Clone number; (b) Plant height; (c) Leaf length; (d) Leaf width; (e) Leaf thickness; (f) Stem diameter.
(TIF)

Table S1 Morphological summary of Changbai Mountain *R. sachalinensis* populations.
(DOC)
