# Supplementary figures and images for: Genetic, Epigenetic, and HPLC Fingerprint Differentiation between Natural and Ex Situ Populations of Rhodiola sachalinensis from Changbai Mountain, China
Source: PLoS One. 2014 Nov 11;9(11):e112869. doi: 10.1371/journal.pone.0112869 (PMC4227887; doi:10.1371/journal.pone.0112869)

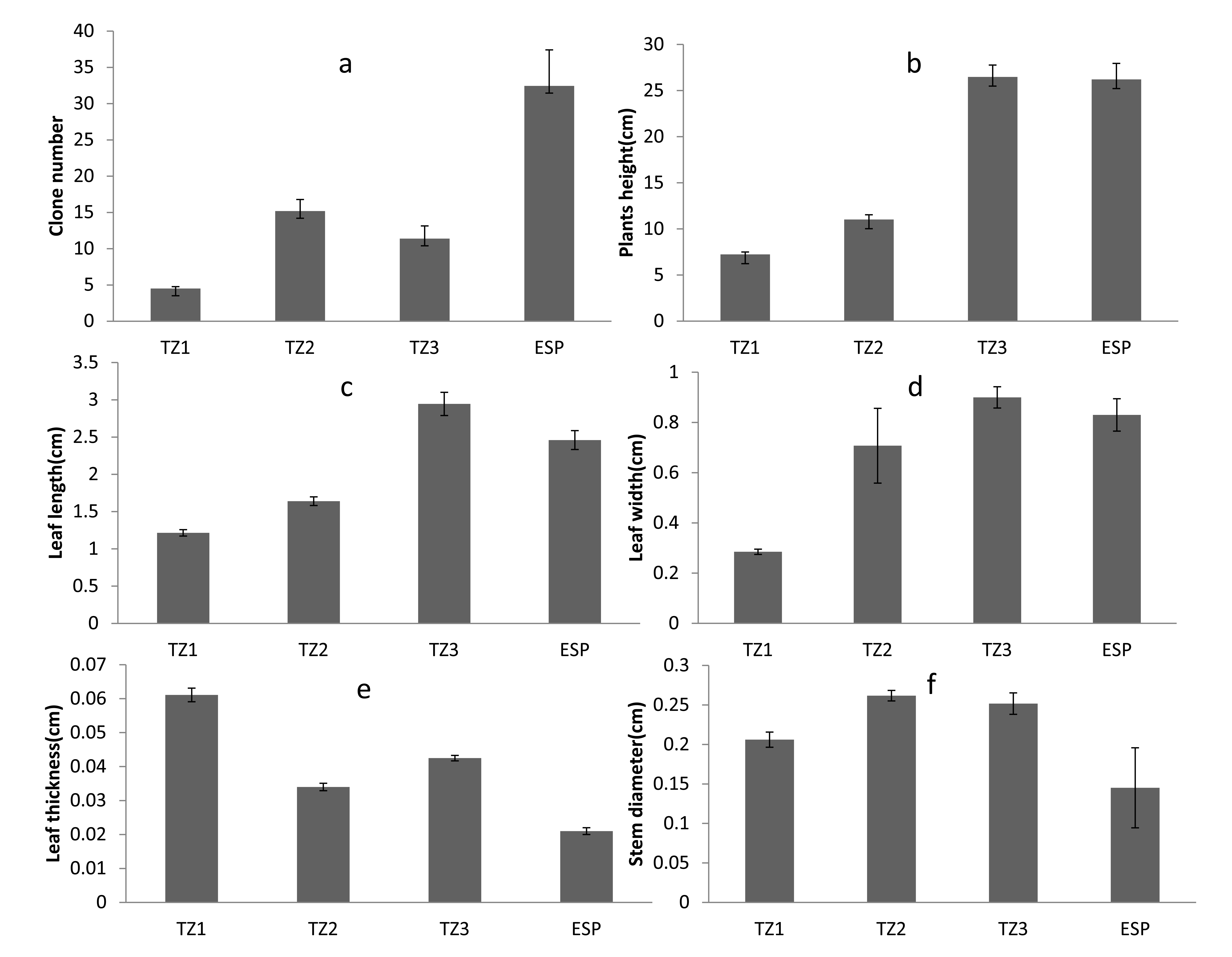

Supplement: Figure S1 — Morphological variation displayed by R. sachalinensis plants from different altitudes on Changbai Mountain. (a) Clone number; (b) Plant height; (c) Leaf length; (d) Leaf width; (e) Leaf thickness; (f) Stem diameter. (TIF) [file pone.0112869.s001.tif]
