# Supplementary material for: Genetic, Epigenetic, and HPLC Fingerprint Differentiation between Natural and Ex Situ Populations of Rhodiola sachalinensis from Changbai Mountain, China
Source: PLoS One. 2014 Nov 11;9(11):e112869. doi: 10.1371/journal.pone.0112869 (PMC4227887; doi:10.1371/journal.pone.0112869)
Supplement: Table S1 — Morphological summary of Changbai Mountain R. sachalinensis populations. (DOC) [file pone.0112869.s002.doc]

**Table S1** Morphological summary of Changbai Mountain *R. sachalinensis* populations

| Population | Clone number | Plants height  (cm) | Leaf length  (cm) | Leaf width  (cm) | Leaf thickness  (cm) | Stem diameter  (cm) |
| --- | --- | --- | --- | --- | --- | --- |
| TZ1 | 4.1500±0.2542 | 7.2300±0.2605 | 1.2150±0.0425 | 0.2850±0.0103 | 0.0611±0.0020 | 0.2061±0.0096 |
| TZ2 | 15.2000±1.5838 | 11.0200±0.5133 | 1.6400±0.0578 | 0.7075±0.1489 | 0.0340±0.0011 | 0.2618±0.0067 |
| TZ3 | 11.4000±1.7402 | 26.4800±1.2918 | 2.9450±0.1557 | 0.9000±0.0423 | 0.0425±0.0008 | 0.2517±0.0136 |
| ESP | 32.4500±4.9622 | 26.2150±1.7339 | 2.4600±0.1266 | 0.8300±0.0645 | 0.0210±0.0010 | 0.1451±0.0507 |

Mean of clone number (No), plant height (H), leaf length (L), leaf width (W), leaf thickness (T) and stem diameter (D) were calculated for different populations of *R. Sachalinensis*.
